# Deletion of CEACAM1 does not affect retinal and choroidal morphology or transcriptome

**DOI:** 10.1007/s00441-026-04087-0

**Published:** 2026-06-26

**Authors:** Laura Hannig, Barbara M. Braunger, Nikolai Kleefeldt, Lea Cagol, Jonathan Zimmermann, Bianka Brunne, Hamza Ahmad, Mario Vallon, Süleyman Ergün, Jost Hillenkamp, Andreas Neueder, Anja Schlecht

**Affiliations:** 1 Institute of Neuroanatomy, University Medical Center Hamburg-Eppendorf, Hamburg, Germany; 2 Department of Ophthalmology, Universitaetsklinikum Würzburg, Würzburg, Germany; 3 Institute of Anatomy and Cell Biology, Julius-Maximilians-University Würzburg, Würzburg, Germany; 4 Institute for Molecular Neurogenetics, Center for Molecular Neurobiology Hamburg, University Clinic Hamburg-Eppendorf, Hamburg, Germany

**Keywords:** CEACAM1, CC1, Retina, Choroid, Homeostasis

## Abstract

CEACAM1 (CC1) is an important mediator of cell proliferation and adhesion and serves as an angiogenic factor through interaction with VEGF. Although the role of CC1 has been extensively studied in organs such as the heart, liver and lung in which CC1 is considered an important regulator of various pathological conditions, little is known about the function of CEACAM1 in the eye. In this study, we investigated the expression and function of CC1 in the retina and choroid of healthy adult mice using immunohistochemistry, fluorescence activated cell sorting (FACS) and RNA sequencing. We found CC1 to be expressed in endothelial and myeloid cells of the retina and the choroid. However, deletion of CC1 did not result in vascular abnormalities of the retina and choroid or changes in retinal myeloid cell morphology and number. Furthermore, the retinal architecture was not affected and morphometric measurements of the thickness of the inner and outer retinal layers were not altered by deletion of CC1. Accordingly, we did not observe structural or transcriptomic changes in the choroid. Our data suggest that the role of CC1 in the adult eye during steady state is attenuated or can be compensated by other molecular mediators. However, one could speculate that CC1 may become functionally relevant during pathological conditions, such as in neovascular eye diseases like proliferative diabetic retinopathy or neovascular age-related macular degeneration.

**Supplementary Information:**

The online version contains supplementary material available at 10.1007/s00441-026-04087-0.

## Introduction

CEACAM1 (CC1), also known as Cluster of differentiation 66a (CD66a), is a transmembrane glycoprotein belonging to the group of carcinoembryonic antigen-related cell adhesion molecules (CEACAM) which is part of the immunoglobulin superfamily (Beauchemin et al. 1999; Beauchemin and Arabzadeh 2013; Rueckschloss et al. 2016). CC1 is expressed in several tissues/organs including the gastrointestinal tract and bone marrow. On a cellular basis, high expression levels for CC1 can be found in epithelial, endothelial and immune cells https://www.proteinatlas.org/ENSG00000079385. The function of CC1 is as diverse as its expression. Under homeostatic conditions, CC1 plays an important role in cell proliferation and cell adhesion (Hammarström 1999). In addition, CC1 can interact with VEGF and thus act as a pro-angiogenic factor: CC1 has been shown to induce the expression of both VEGF and VEGFR2 and, conversely, the presence of VEGF can also increase CC1 expression (Ergün et al. 2000; Kilic et al. 2005). This reciprocal relationship creates a pro-angiogenic microenvironment (Götz et al. 2024). In terms of pathogenesis CC1 has been implicated in cancer development and progression. Elevated levels of CC1 have been detected in colorectal cancer and other cancer entities and have been corelated with shorter patient survival (Kang et al. 2007; Thöm et al. 2009; Ieda et al. 2011; Yang et al. 2015).

The function of CC1 in the eye has been scarcely investigated to date and remains poorly understood. Furthermore, the specific expression pattern of CC1 especially in the retina and choroid are not yet defined. To date, there is one publication focusing on the role of CC1 in a mouse model of pathological angiogenesis during development: CC1-deficient mice showed exacerbated retinal neovascularization and increased tuft formation, concomitant with delayed revascularization and vessel maturation (Ludewig et al. 2014).

To further unravel the role of CC1 in retina and choroid during adulthood, we studied CC1 expression in these tissues. Furthermore, we used CC1 knockout mice and compared those with wild type littermates regarding transcriptome, structure and immune cell abundance and morphology. Using this approach, we found a strong CC1 protein expression in endothelial cells and microglia. The deletion of CC1 did not lead to relevant changes in the transcriptome, nor did we observe changes in the retinal and choroidal architecture. Likewise, there were no differences in the number or reactivity of microglial cells depending on CC1 expression. On the basis of these findings, we assume that CC1 does not play a relevant role during tissue homeostasis despite its prominent expression in endothelial and immune cells. However, it is conceivable that, in the event of damage—particularly involving endothelial and/or immune cells—CC1 could play a significant role in the eye during pathological conditions.

## Results

### Expression of CEACAM1 in the murine retina and choroid

**Fig. 1:**
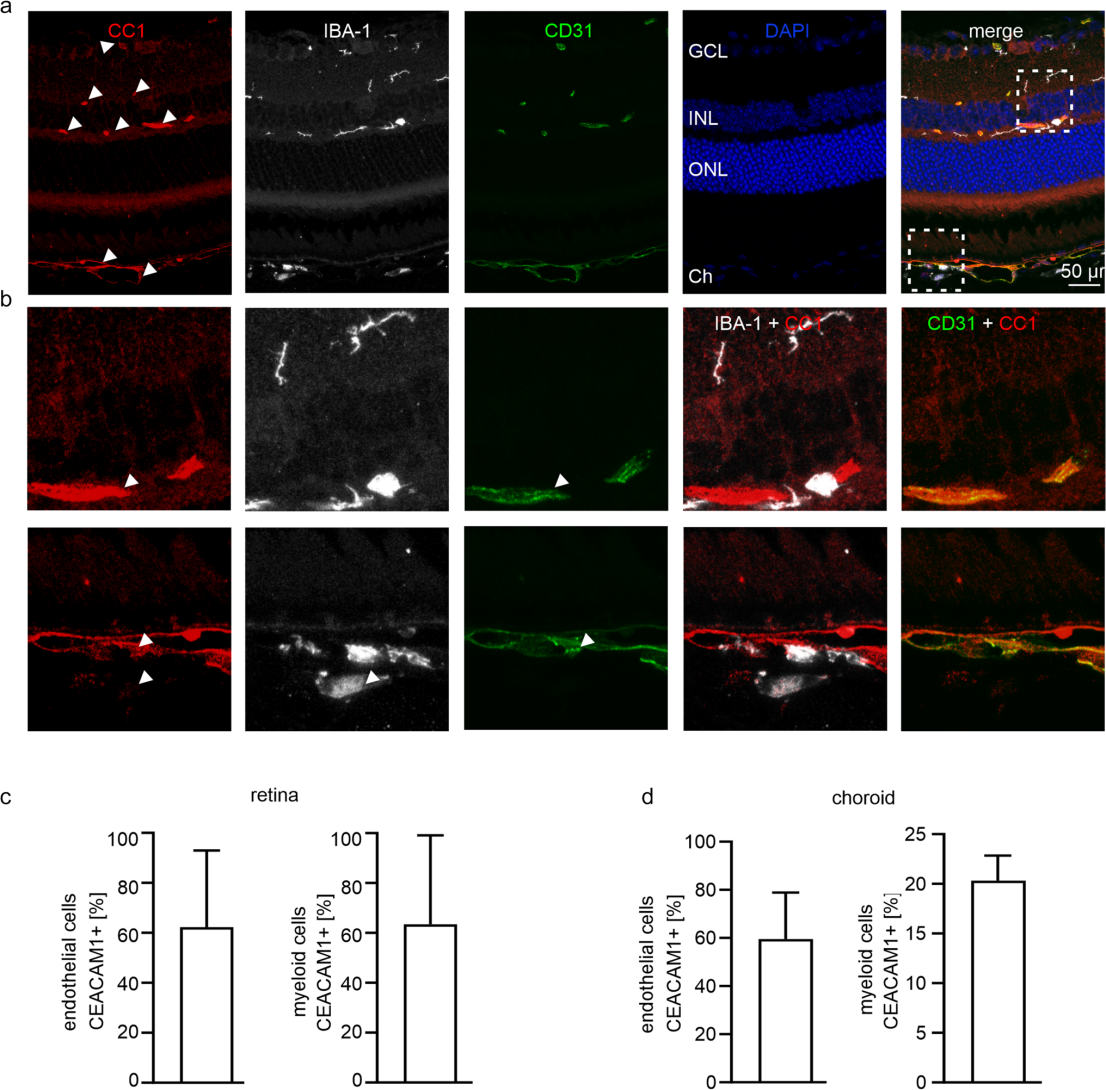
CEACAM1 expression in retina and choroid. **a**: CEACAM1 immunoreactivity (red) is detectable in the ganglion cell layer, the inner and outer plexiform layer and choroid (white arrowheads), endothelial cells are labeled with CD31 (green), myeloid cells are labeled using IBA-1 (white). The cell nuclei are stained with DAPI (blue). **b**: Higher magnification of boxed areas from A. (upper panel: higher magnification of retinal area, lower panel: higher magnification of choroidal area). Arrowheads points towards areas with colocalization of CC1 with either CD31 or IBA-1C, **d**: Flow cytometry revealed CEACAM1 expression in endothelial cells and myeloid cells of the retina (C, *n* = 10) and the choroid (D, *n* = 8) of wild type*.* Data are means ± SD. GCL = ganglion cell layer, IPL = inner plexiforme layer, INL = inner nuclear layer, OPL = outer plexiforme layer, ONL = outer nuclear layer, Ch = choroid

As there is, to the best of our knowledge, a paucity of data regarding the role and expression of CEACAM1 (CC1) in ocular tissues, we initially assessed CC1 protein expression in adult murine eyes using immunohistochemical staining and fluorescence-activated cell sorting (FACS). In the retina, we observed pronounced CC1 immunoreactivity in the ganglion cell layer, as well as in the inner and outer plexiform layers (Fig. 1a and b). Given that these regions coincide with the localization of intraretinal vasculature, we performed co-immunolabeling with CD31, a marker for endothelial cells and IBA-1, a marker for myeloid cells (Fig. 1a and b). CEACAM1 expression was detected in CD31 positive endothelial cells of the retina and choroid and partly in IBA-1 positive cells (Fig. 1b). This was further investigated by FACS analysis (Fig. 1c). Since the retinal regions exhibiting CC1 expression are also known to harbor myeloid cells, we analyzed this population for CC1 expression using FACS. Our results demonstrated that CD11b⁺ myeloid cells also express CC1 (Fig. 1c). In addition to the retinal expression of CC1, we also detected robust immunoreactivity in the choroidal tissue (Fig. 1a), where, similar to the retina, CC1 expression could be attributed predominantly to endothelial and myeloid cells (Fig. 1d).

### Deletion of CEACAM1 does not affect retinal vasculature or myeloid cell characteristics

**Fig. 2:**
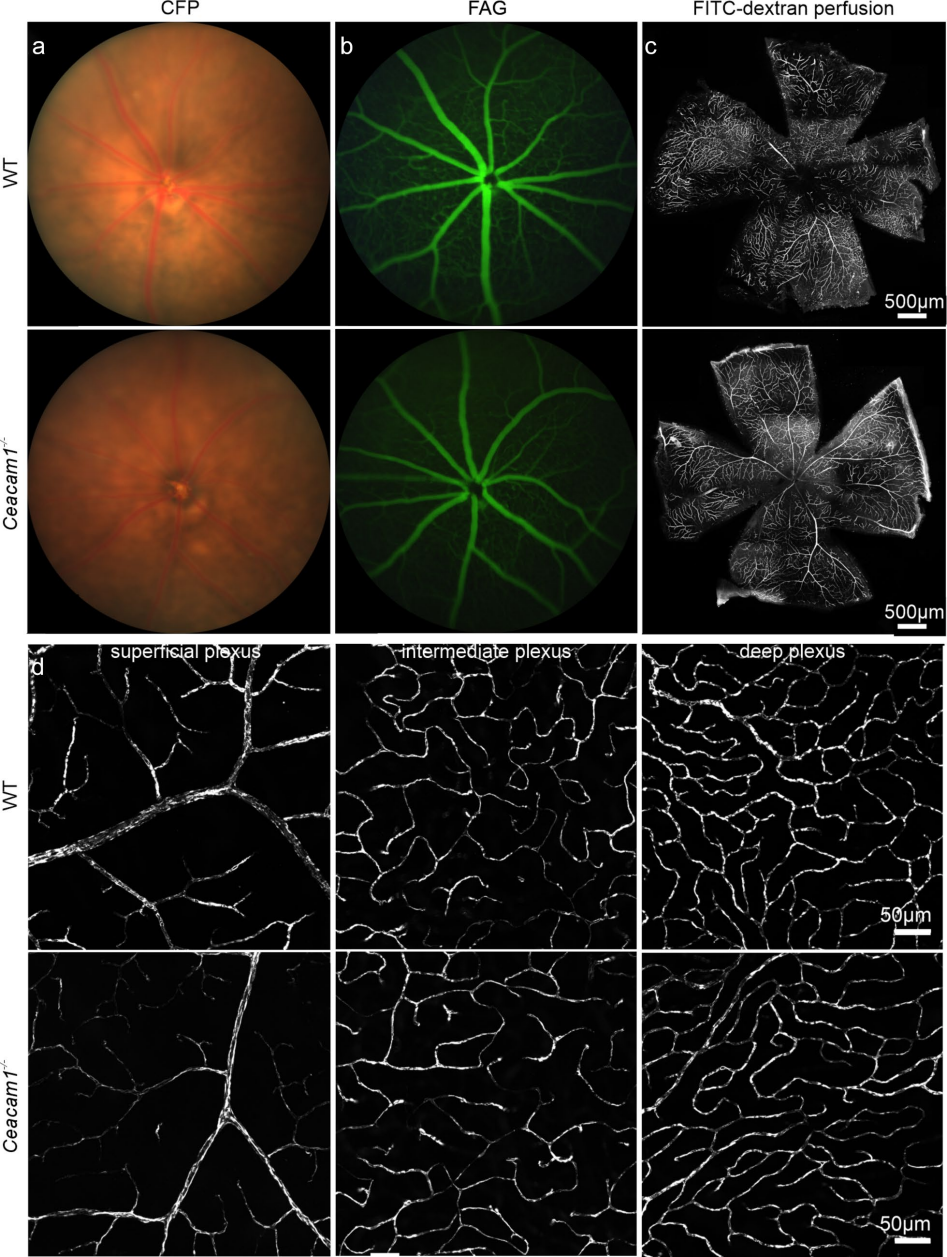
Retinal vasculature. **a**, **b**: In vivo imaging of the retinal vessels using color fundus photography (CFP) (A) and fluorescein angiography (FAG) (B). **c**, **d**: Ex vivo imaging using fluorescein isothiocyanate (FITC)-dextran perfusion

To assess the functional relevance of CC1 expression in the retina—particularly given its prominent expression in endothelial cells—we used *Ceacam1*^−/−^ mice and performed *in vivo* color fundus photography and angiography (validation of successful deletion of CC1 is shown in Supplementary Fig. 1). In both, *Ceacam1*^−/−^ and wild type littermates, we found a regular fundus with no signs of abnormalities (Fig. 2a). Fluorescein angiography revealed evenly distributed, radiating vessels originating from the optic nerve head for both mouse groups. No evidence of leakage was detectable, neither in the wild type, nor in *Ceacam1*^−/−^ mice suggesting an intact blood-retinal barrier (Fig. 2b). To enable a more detailed morphological assessment of the retinal vasculature, we performed *in vivo* perfusion with FITC-dextran followed by preparation of retinal flatmounts (Fig. 2c and d) allowing for the visualization of the three vascular plexus. In wild type mice, the superficial vascular plexus exhibited its characteristic arrangement of prominent vessels emerging from the optic nerve head, while both, the intermediate and deep vascular plexus showed the expected fine network (Fig. 2d). Comparable vascular patterns were observed in *Ceacam1*^−/−^ mice. We observed a continuous and well-organized vascular architecture, with no signs of vessel rarefaction, abnormal branching, or microaneurysms (Fig. 2d). Therefore, we conclude that the deletion of CC1 does not affect the structural integrity or organization of the retinal vasculature under physiological conditions.

**Fig. 3:**
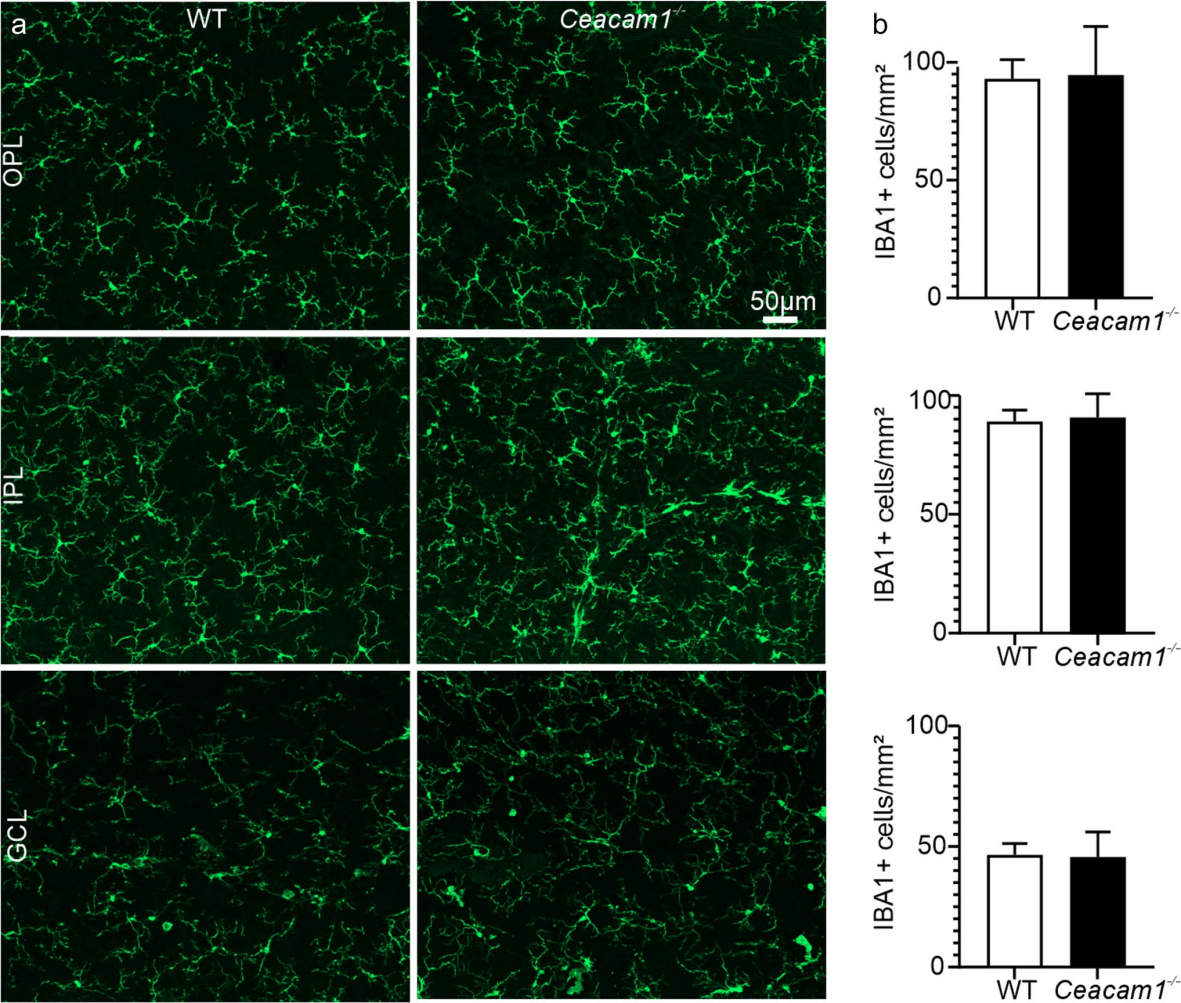
Myeloid cell abundance and morphology. **a**: Visualization of myeloid cells by immunohistochemical staining for ionized calcium-binding adapter molecule 1 (IBA1), shown for the outer plexiform layer (OPL), the inner plexiform layer (IPL) and the retinal ganglion cell layer (GCL). **b**: Quantification of IBA1 + cells in the GCL, IPL and OPL (wild type *n* = 4, *Ceacam1*^*−/−*^ (*n* = 7). Mean ± SD is shown

Given the detected expression of CC1 in retinal myeloid cells, we further characterized this cell population in both wild type and *Ceacam1*^−/−^ mice to assess potential differences (Fig. 3a and b). The morphological appearance of these cells was comparable in both genotypes: They exhibited the typical ramified, dendritic morphological characteristic for resting myeloid cells in the retina. No signs of hypertrophy, process retraction, or clustering were observed (Fig. 3a). We furthermore quantified myeloid cells in all three compartments, where retinal myeloid cells typically reside in the retina. Cell counts revealed no significant difference in the number of myeloid cells between wild type and *Ceacam1*^−/−^ mice (Fig. 3b). These findings suggest that the absence of CC1 does not impact the number or morphology of retinal myeloid cells.

### Deletion of CEACAM1 does not affect retinal or choroidal structure

**Fig. 4:**
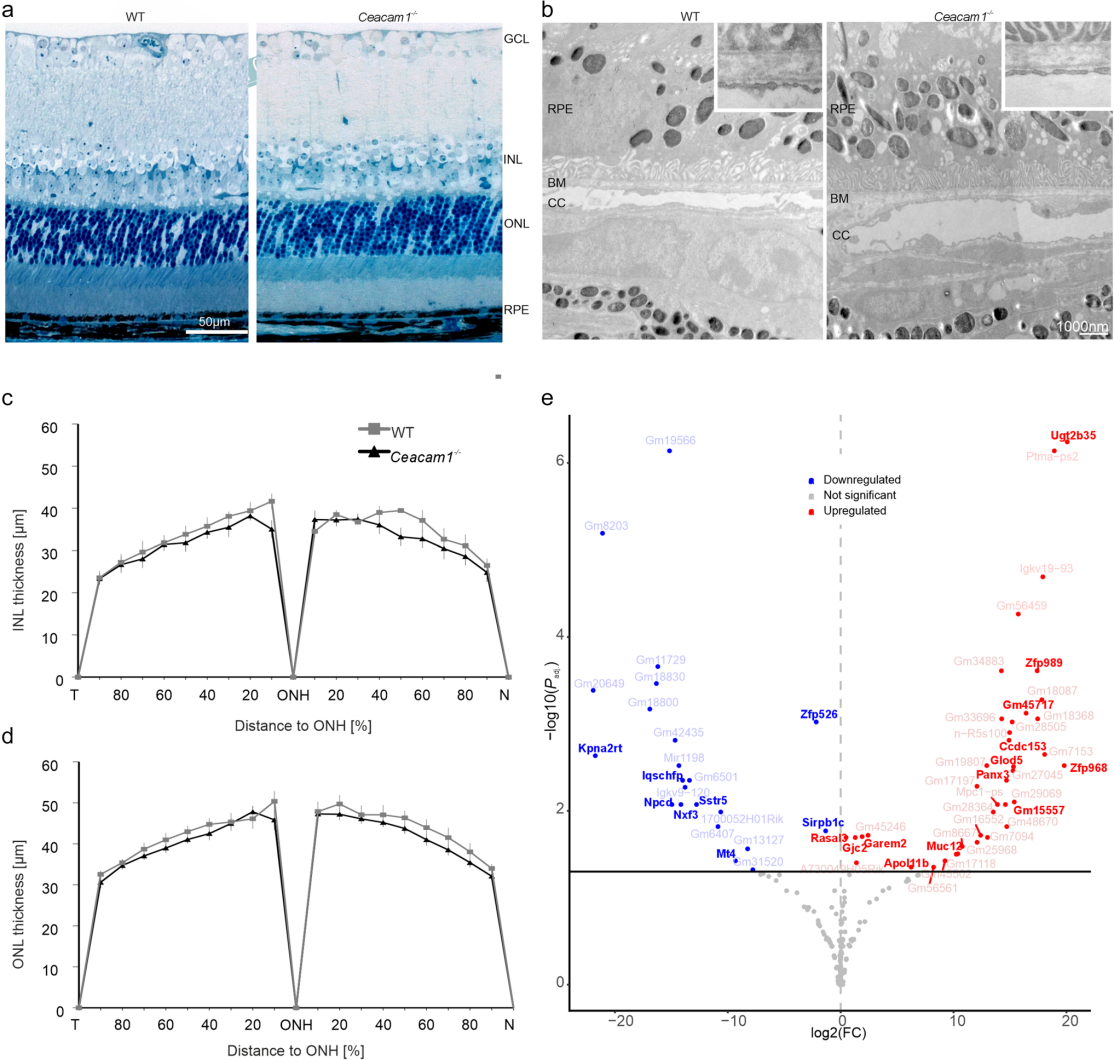
Retinal and choroidal structure. **a**: Semithin sections stretching through the optic nerve head. **b**: Electron microscopy of the RPE, Bruch’s Membrane and choroid. Boxed area shows fenestrations of the endothelium RPE = retinal pigmented epithelium; BM = Bruch’s membrane, CC = choriocapillaris. **c**, **d**: Morphometric analyses of the thickness of the inner (C) and outer nuclear layer (D) showed no significant differences between wild type (*n* = 5) and *Ceacam1*^*−/−*^ (*n* = 7) mice (p > 0.05), GCL = ganglion cell layer, INL = inner nuclear layer, ONL = outer nuclear layer, RPE = retinal pigment epithelium, T = temporal, ONH = optic nerve head, *N* = nasal. Data are means ± SEM. **e**: Volcanoplot illustrating differentially expressed genes (DEGs, p. adj. ≤ 0.05) between choroidal tissue from wild type (*n* = 4) and *Ceacam1*.^−/−^ (*n* = 4) mice. DEG are colored in blue (downregulated) and red (upregulated)

In the final step of our analysis, we examined the retinal and choroidal tissue architecture using semithin sections to assess potential structural alterations at high resolution. We found a regular organization of the retina as well as of the choroid in both, wild type and *Ceacam1*^−/−^ mice: The retinal layers were well defined, with no evidence of disorganization (Fig. 4a). To detect even subtle morphological changes and screen for neurodegeneration, we performed morphometric measurements of the thickness of the inner nuclear layer (INL) and outer nuclear layer (ONL) followed by statistical analysis. These analyses revealed a largely comparable ONL and INL thickness between wild type and *Ceacam1*^−/−^ mice (Fig. 4c and d). The choroid also appeared structurally intact, no abnormalities were observed: The vessels were evenly distributed, with no signs of vessel dilation, or abnormal thickening (Fig. 4a and b). Since the choroid is the densest vascular network in the human body, we additionally performed electron microscopy and RNAseq analyses to ensure that we did not overlook subtle changes in regard to CC1 expression. We could not detect any genotype-dependent structural changes of the retinal pigment epithelium (RPE), the adjacent Bruch’s Membrane or the fenestration of the endothelium (Fig. 4b). Consistent with the histological findings, RNA-Seq data revealed no major differences in gene expression profiles of the choroid in *Ceacam1*^−/−^and wild type mice: We found a high degree of transcriptional similarity between *Ceacam1*^−/−^ and wild type choroideae. Of all the detected genes, only 61 were differentially expressed (adjusted *p* < 0.05, Fig. 4e). Of these, merely 21 encoded proteins, with 8 genes being downregulated and 13 upregulated in the choroid of *Ceacam1*^−/−^ mice (non-protein coding genes are shown in a lighter color). Gene ontology (GO) enrichment analysis performed on this set of protein-coding genes did not yield any significant results. In summary, these findings indicate that deletion of CC1 does not result in alterations in either the retina or the choroid under physiological conditions.

## Discussion

To the best of our knowledge, this study is the first one to characterize the spatial distribution of CEACAM1 expression and its function in the adult, healthy retina and choroid. We observed that CC1 is predominantly expressed in endothelial cells and myeloid cells of both the retina and the choroid of adult healthy mice.

### Expression of CC1 in the vascular endothelial cells

CC1 expression in vascular endothelial cells has been described in other organs, such as the lung, liver or testis, but interestingly in these organs, high CC1 expression appears to be associated with pathological conditions (Lauke 2004; Thöm et al. 2009; Muturi et al. 2023). In these organs, CC1 expression is low or absent under physiological conditions. However, with the development of pathologies, such as seminomas or carcinomas, CC1 is upregulated in endothelial cells (Lauke 2004; Thöm et al. 2009; Muturi et al. 2023). Similar results have been reported for brain endothelial cells: Basal CC1 expression in the steady state is replaced by strong upregulation of CC1 after stroke (Ludewig et al. 2013). In general, an upregulation of CC1 can be observed in active endothelia, e.g. in tumor growth, wound healing processes, but also in physiological angiogenesis during development (Sawa et al. 1994; Ergün et al. 2000; Ling et al. 2015). In this context, CC1 can act as a pro-angiogenic factor by interacting with VEGF: CC1 has been shown to induce the expression of both VEGF and VEGFR2 and, conversely, the presence of VEGF can also increase CC1 expression (Ergün et al. 2000; Kilic et al. 2005) leading to a pro-angiogenic microenvironment (Götz et al. 2024). This bidirectional relationship could explain the strong CC1 expression observed in the healthy adult mouse retina and choroid. The retinal pigment epithelium (RPE) secretes substantial amounts of VEGF during both embryonic development and adulthood (Ford et al. 2011). In adulthood, sustained VEGF expression is essential for maintaining the fenestrated structure of the choriocapillaris (Saint-Geniez et al. 2009; Kurihara et al. 2012). It is likely that the high VEGF levels secreted by the RPE contribute to the persistent expression of CC1 in the choroidal endothelium.

The function of CC1 in the vascular endothelium appears to be rather different depending on whether physiological or pathological conditions dominate. For example, deletion of CC1 in the brain in healthy mice does not lead to vascular changes such as leakiness or edema (Ludewig et al. 2013). This is consistent with our observations in the retina or choroid of CC1-deficient mice. However, during experimental induced stroke CC1-deficient mice revealed a more severe blood brain barrier breakdown concomitant with increased cerebral edema (Ludewig et al. 2013). It is tempting to speculate that in ocular diseases associated with a breakdown of the blood-retinal barrier (BRB), such as age-related macular degeneration (outer BRB) or diabetic retinopathy (inner BRB), CC1 deficiency may also result in a deteriorated outcome.

### Expression of CC1 in immune cells

Other than endothelial cells, immune cells have also been reported in the literature to be a potent source of CC1 as summarized by Gray-Owen and Blumberg (Gray-Owen and Blumberg 2006): CEACAM1 expression in immune cells varies by cell type and activation status. While neutrophils, B cells and dendritic cells can constitutively express CC1, T cells display rather low CC1 levels when resting, with rapid surface mobilization upon activation by mitogens or cytokines, such as IL-2, IL-7, IL-15 (Moller et al. 1996; Donda et al. 2000; Boulton and Gray-Owen 2002). Natural killer (NK) cells do not express CC1 at rest but NK cells can rapidly upregulate it following stimulation. In this context CC1 has the ability to modulate immune responses (Gray-Owen and Blumberg 2006). In addition to its immunomodulatory functions, CEACAM1 has also been described as a receptor for several viral, bacterial, and fungal pathogens, including mouse hepatitis virus (MHV) in mice (Taguchi and Hirai-Yuki 2012; Kim et al. 2019). While microglial CC1 expression has not been described in the brain under homeostatic conditions or in pathological conditions such as stroke (Ludewig et al. 2013), we see pronounced expression of CC1 in retinal microglia or in myeloid cells in the choroid even without experimental manipulation. However, deletion of CC1 did not result in altered myeloid cell characteristics, neither in terms of cell morphology nor cell numbers, suggesting that the function of CC1 in resting retinal microglia is negligible.

### Implications of CC1 for ocular homeostasis and disease

Beyond its established roles in angiogenesis, CEACAM1 has also been implicated in both aging-related and inflammation-dependent cellular processes: Previous studies have shown that CEACAM1 expression can be modulated by pro-inflammatory cytokines, including TNF (Götz et al. 2025), and that increased CEACAM1 signaling has been associated with vascular aging and chronic inflammatory conditions (Kleefeldt et al. 2019; Götz et al. 2025). These findings suggest a role for CEACAM1 in inflammatory signaling, endothelial dysfunction, and tissue remodeling under pathological conditions. Given that chronic low-grade inflammation and age-associated tissue degeneration are central features of many retinal and choroidal diseases, these context-dependent functions of CEACAM1 may be particularly relevant in the diseased eye. In addition, the involvement of CEACAM1 in endothelial and barrier-associated responses (Kleefeldt et al. 2019) further supports its potential contribution to inflammatory microenvironmental changes during ocular disease conditions. Age-related macular degeneration (AMD), in particular its neovascular from, may represents such a condition. Neovascular AMD is one of the most common causes of blindness in the elderly population with its main feature being the development of choroidal neovascularization (CNV), concomitant with a breakdown of the outer blood retinal barrier (Guyer et al. 1986; Congdon et al. 2004; Wong et al. 2008). CNV are defined by the formation of new blood vessels that originate from the choroidal vasculature and grow into the subretinal space. This is usually accompanied by edema formation and hemorrhage, leading to rapid, severe and irreversible vision loss (Sulzbacher et al. 2017). Myeloid cells, such as resident microglia or infiltrating macrophages have been shown to modulate disease progression (Schlecht et al. 2020, 2021; Wolf et al. 2020) suggesting a strong endothelial-myeloid cell interaction during AMD. The dual cellular compartments where CEACAM1 is expressed in the eye, align precisely with these two communication partners during AMD. It is therefore reasonable to assume that CEACAM1 may well act as a potential mediator of neovascular age-related macular degeneration.

## Conclusion

In summary, this study shows that endothelial and myeloid cells are the predominant CEACAM1 expressing cells in the healthy retina and choroid. Deletion of CC1 did neither change vascular integrity, myeloid cell number or neuronal layer thickness in the retina nor choroidal structure or transcriptome. Our findings therefore suggest that the role of CC1 in the adult steady state is attenuated or can be compensated by other molecular mediators. However, one could speculate that CC1 could become functionally relevant in ocular diseases such as in neovascular age-related macular degeneration in which endothelial cells and myeloid cells are central regulators.

## Materials and methods

### Mice

All procedures conformed to the tenets of the National Institutes of Health Guidelines on the Care and Use of Animals in Research, the EU Directive 2010/63/E. The study is reported in accordance with ARRIVE guidelines. For experiments mice (obtained from the Institute of Anatomy and Cell Biology, Wuerzburg, Germany) carrying heterozygous deletion of Exon1 and 2 of *Ceacam1* gene (Leung et al. 2006) were mated to generate homozygous *Ceacam1* knockout mice and wild type control littermates. Mice were bred on a C57BL/6J background and kept in cyclic light (12 h on/12 h off, lights on at 7 am, light intensity approx. 400 lx). Unless otherwise stated, mice were 6–8 weeks old and weighed 15–25 g. Both sexes were used for experiments and all mice were tested negatively for rd8 mutation.

### *In vivo* imaging

Fundus morphology, retinal structure and vasculature were investigated using fundus photography and fundus fluorescein angiography (FFA), as previously described (anesthesia: 100 mg ketamine/5 mg xylazine per kg bodyweight) (Lange et al. 2012). Fundus photography and FFA were performed using a Micron IV retinal microscope (Phoenix Technology Group, Campbell, CA, USA) and the StreamPix software v8.4.0 (Norpix Inc., Montreal, Canada) as described in (Zhang et al. 2021) For FFA, 10% sodium fluorescein (Alcon, Freiburg, Germany) was diluted to a concentration of 50 μL/mL in 0.9% sodium chloride for injection and administered intraperitoneally (2 μL/g). Ninety seconds after dye injection, the angiograms were recorded (Zhang et al. 2021).

### FITC Dextran perfusion

Before dextran perfusion, mice were killed by CO_2_ euthanasia. Afterwards, mice were perfused through the left ventricle with 1 mL of PBS containing 50 mg fluorescein isothiocyanate (FITC)–dextran (mol. wt., 2000 kDa; TdB Consultancy, Uppsala, Sweden) (Schlecht et al. 2017). The eyes were enucleated and fixed in 4% paraformaldehyde (PFA) for 2 h.

### Fluorescence microscopy

Prior to COL IV (2150–1470, Biorad, Germany 1:40), CD31 (77699, Cell Signaling, Danvers, MA, USA 1:200), IBA-1 (234009, Synaptic Systems, Göttingen, Germany, 1:500) and CEACAM1 (CC1 mAb, 1:100 (Williams et al. 1991; Rovituso et al. 2016)) staining, eyes were fixed for 4 h in 4% PFA, and embedded in paraffin, according to standard protocols. Paraffin sections (6 μm thick) were deparaffinized and washed using water. Sections were pretreated with boiling citrate buffer (1 × 10 min, pH 6). CEACAM1 staining was performed in combination with M.O.M. kit (Vector Laboratories, Newark, CA, USA). Images of paraffin sections were taken using an Axio Imager Z1 fluorescent microscope and Axiovision software v4.8 (Zeiss, Jena, Germany) or Leica TCS SP5 confocal microscope (Leica Camera AG, Wetzlar, Germany).

For flatmount staining, mice were perfused with phosphate-buffered saline (PBS) and 4% PFA, eyes were fixed in 4% PFA for 1 h on ice and processed for flatmounts, as previously described (Lange et al. 2012; Wang et al. 2013). Anti-mouse IBA-1 (Wako, 019–19741, Osaka, Japan) was added in a 1:500 dilution at 4 °C for 2 nights. A secondary antibody was applied in a dilution of 1:500 (Alexa Fluor® 488, Thermo Fisher Scientific, Waltham, MA, USA) overnight at 4 °C. Images of the mid-periphery of flatmounts were taken using an Axio Imager Z1 fluorescent microscope and appropriate Axiovision software v4.8 (Zeiss, Jena, Germany).

### Light microscopy and spider diagrams

Eyes were carefully enucleated and fixed for 24 h in Ito’s fixative (Braunger et al. 2013a). As described in Bielmeier et al. (2022), the eyes were marked with a thin, short metal needle at the superior limbus and embedded in Epon (Serva, Heidelberg, Germany). Semithin meridional sections (in nasal-temporal orientation) of 1.0 μm thickness were cut stretching through the optic nerve head (ONH) and the pupil. Sections were stained according to the Richardson’s protocol (Richardson et al. 1960) and images taken using an Axio Imager Z1 light/fluorescent microscope (Zeiss, Jena, Germany). The thickness of the outer nuclear layer (ONL) was measured at nine equidistant loci along the circumference of each hemisphere as described in (Braunger et al. 2013a, b, 2015; Boneva et al. 2016). The means and corresponding standard errors of the mean (SEM) were calculated for each measure point and the results were plotted as spider diagram using GraphPad Software, Version 6.0 (La Jolla, CA, USA).

### Fluorescence-activated cell sorting

Mice were euthanized by CO2 exposure followed by cardiac perfusion with 10 ml ice-cold PBS. For further analysis, eye cups of PBS-perfused mice were dissected to separate the retina from underlying RPE/choroid/sclera. For simplicity, the RPE/choroid/sclera complex will be referred to as 'chroid' in the following. First, the tissues were mechanically treated by repeatedly aspirating them with a pipette and dispensing them again. Then retinal or choroidal tissue was dissociated by resuspension followed by digestion using collagenase IV (100 U/µl Cell Systems, Troisdorf, Germany, LS004188) and DNase (10 U/µl Cell Systems, Troisdorf, Germany, LS006326) in DMEM/HEPES (37°C 30-45min) and then filtered. Dead cells were excluded by incubation in fixable viability dye 456 (1:1000, BD Horizon, Heidelberg, Germany, #562247) on ice for 15 min in the dark. Anti-CD16/CD32 Fc block (Invitrogen, Carlsbad, CA, USA #14–0161-82) was performed on ice for 15 min in the dark (1:200) to avoid unspecific binding. Afterwards, cells were stained with antibodies against CEACAM1 (1:100 mouse antibody (Williams et al. 1991; Rovituso et al. 2016)), CD11b (Invitrogen, Carlsbad, CA, USA, 25–0112-82 1:200), CD31 (Invitrogen, Carlsbad, CA, USA, 25–0311-82 1:200) on ice for 30 min in the dark. Following staining, cells were washed and analyzed using FACS Canto (BD Biosciences, Heidelberg, Germany) and flowjo analysis software version 9 (Waters Biosciences, Ashland, OR, USA).

### RNA extraction

Total RNA was extracted from mouse choroid tissue preserved in RNAlater buffer using the *RNeasy Micro Kit* (QIAGEN, Hilden, Germany), following the manufacturer’s instructions for “Purification of total RNA from animal and human tissue.” In short, samples were kept in RNAlater and transported at 2–8 °C. The tissue was centrifuged at 5,000 × g for 5 min, after which the preservative was removed. The pellet was then lysed and homogenized in 350 µl of RLT buffer supplemented with 1% β-mercaptoethanol, using Precellys CK14 ceramic beads (one 15 s cycle at 5500 rpm) in a Precellys 24 Homogenizer (Bertin Corp., Rockville, MD, USA). The homogenate was centrifuged at 20,000 × g for 2 min, and 350 µl of the cleared supernatant was transferred into a fresh tube. An equal volume of 70% ethanol was added, and the mixture was applied to an RNeasy MinElute spin column. After on-column DNase treatment and several wash steps, RNA was eluted in 14 µl of nuclease-free water. The quality and integrity of the isolated RNA were determined using the Agilent 2100 Bioanalyzer together with the RNA 6000 Nano LabChip kit (Agilent, Palo Alto, CA, USA).

### RNA sequencing

Library preparation and RNA sequencing were performed following the *SMART-Seq mRNA LP User Manual* (Takara Bio, Inc., Kusatsu, Japan), the *Illumina NextSeq 2000 Sequencing System Guide* (Illumina, Inc., San Diego, CA, USA), and the *KAPA Library Quantification Kit Protocol* (Roche Sequencing Solutions, Inc, Pleasanton, CA, USA.). Briefly, 10 ng of total RNA was used for first-strand cDNA synthesis. Double-stranded cDNA was amplified by 8 cycles of LD-PCR and purified with magnetic beads. Approximately 500 pg of cDNA was enzymatically fragmented, ligated to stem-loop adapters, and PCR-amplified (14 cycles) with unique dual indexes (UDIs). Libraries were purified, quantified with the KAPA kit, and pooled in equimolar amounts. Sequencing was performed on an Illumina NextSeq 2000 using a 100-cycle P3 Flow Cell in paired-end dual-index mode. Image analysis and base calling were conducted with RTA v3.10.30, and.cbcl files were converted to.fastq using bcl2fastq v2.20.

### Bioinformatics

FastQ files were quality checked with FastQC v0.11.5 (Andrews 2010). All files passed QC. The reads were pseudo-aligned against Ensembl *Mus musculus* GRCm39 release vM35 and quantified using salmon v1.10.3 (Patro et al. 2017). Samples were screened for outliers using a combined PCA and clustering analysis. To this end, counts were imported using tximport v1.32.0 (Soneson et al. 2015) into DESeq2 v1.44.0 (Love et al. 2014) and an intercept matrix of all samples was computed. This matrix was corrected for sex of the mice using limma v3.60.6 (Ritchie et al. 2015). A sample was defined as an outlier if it was outside a 68% probability ellipse in PCA analysis (Marini and Binder 2019) and was outside a −2.5 standardized connectivity cutoff of an Euclidean distance matrix of all samples (Oldham et al. 2012). This procedure identified 1 outlier sample, which was removed from the subsequent evaluation. Final group distribution for the evaluation was as follows: Wild type *N* = 4; *Ceacam1*^−/−^ *N* = 4. Transcriptional dysregulation was computed as above with the exception of using a design matrix that included sex of the mice as a covariate and genotype as the variable of interest. Ashr was used as the fold change shrinkage estimator (Stephens 2017). DESeq2 analysis files are available as supplementary information files Scripts are available upon request.

### Statistical analysis

Statistical analysis (t-test) was performed using the software GraphPad Prism (GraphPad Software, Version 6.0, La Jolla, CA, USA). Differences were considered to be statistically significant for *P*-values < 0.05.

## Supplementary Information

Below is the link to the electronic supplementary material.

Supplementary Figure 1: Deletion of CC1 in retina and choroid. A, B: Retinal (A) and choroidal (B) mRNA expression of Ceacam1 in 2-4 month-old wild type and Ceacam1-/- mice. Data are means ± SD. (retina: wild type n = 3 and Ceacam1-/- n = 5; choroid: wild type n = 6 and Ceacam1-/- n = 6), *** p ≤ 0.001. C : CC1 (green, arrows) and COL IV (red, arrowheads) double labelling of 3 months old wild type and Ceacam1-/- eyes. Nuclei are DAPI-stained (blue). GCL = ganglion cell layer, INL = inner nuclear layer, ONL = outer nuclear layer. (PNG 689 KB)

High Resolution Image (TIF 6.68 MB)

Supplementary Figure 2: Representative FACS plots for gating strategy and isotype controls. A representative flow cytometry gating strategy for analyzing CEACAM1 expression in myeloid and endothelial cells of the retina (A-D) and choroid (E-H). First, cells were identified based on FSC-A/SSC-A, then singlets were selected using FSC-H/FSC-A, and live cells were gated using live/dead cell staining. Next, endothelial cells (CD31+) (A, E) or myeloid cells (CD11b+) (C, G) were identified, and CEACAM1 expression was analyzed in comparison to the respective isotype control. The histograms show the distribution of CEACAM1-positive endothelial (B, F) or myeloid cells (D, H). (PNG 674 KB)

High Resolution Image (TIF 17.1 MB)

## Data Availability

RNA Sequencing Data is available from GEO with the accession number GSE307521.

## References

[CR1] Andrews S (2010) FastQC: a quality control tool for high throughput sequence data. Available only online at: http://www.bioinformatics.babraham.ac.uk/projects/fastqc

[CR2] Beauchemin N, Arabzadeh A (2013) Carcinoembryonic antigen-related cell adhesion molecules (CEACAMs) in cancer progression and metastasis. Cancer Metastasis Rev 32:643–671. 10.1007/s10555-013-9444-6 23903773 10.1007/s10555-013-9444-6

[CR3] Beauchemin N, Draber P, Dveksler G et al (1999) Redefined nomenclature for members of the carcinoembryonic antigen family. Exp Cell Res 252:243–249. 10.1006/excr.1999.4610 11501563 10.1006/excr.1999.4610

[CR4] Bielmeier CB, Schmitt SI, Kleefeldt N et al (2022) Deficiency in retinal TGFβ signaling aggravates neurodegeneration by modulating pro-apoptotic and MAP kinase pathways. Int J Mol Sci 23:2626. 10.3390/ijms23052626 35269767 10.3390/ijms23052626 PMC8910086

[CR5] Boneva SK, Groß TR, Schlecht A et al (2016) Cre recombinase expression or topical tamoxifen treatment do not affect retinal structure and function, neuronal vulnerability or glial reactivity in the mouse eye. Neuroscience 325:188–201. 10.1016/j.neuroscience.2016.03.050 27026593 10.1016/j.neuroscience.2016.03.050

[CR6] Boulton IC, Gray-Owen SD (2002) Neisserial binding to CEACAM1 arrests the activation and proliferation of CD4+ T lymphocytes. Nat Immunol 3:229–236. 10.1038/ni769 11850628 10.1038/ni769

[CR7] Braunger BM, Ohlmann A, Koch M et al (2013a) Constitutive overexpression of Norrin activates Wnt/β-catenin and endothelin-2 signaling to protect photoreceptors from light damage. Neurobiol Dis 50:1–12. 10.1016/j.nbd.2012.09.008 23009755 10.1016/j.nbd.2012.09.008

[CR8] Braunger BM, Pielmeier S, Demmer C et al (2013b) TGF-β signaling protects retinal neurons from programmed cell death during the development of the mammalian eye. J Neurosci 33:14246–14258. 10.1523/JNEUROSCI.0991-13.2013 23986258 10.1523/JNEUROSCI.0991-13.2013 PMC6618509

[CR9] Braunger BM, Leimbeck SV, Schlecht A et al (2015) Deletion of ocular transforming growth factor β signaling mimics essential characteristics of diabetic retinopathy. Am J Pathol 185:1749–1768. 10.1016/j.ajpath.2015.02.007 25857227 10.1016/j.ajpath.2015.02.007

[CR10] Congdon N, O’Colmain B, Klaver CCW et al (2004) Causes and prevalence of visual impairment among adults in the United States. Arch Ophthalmol 122:477–485. 10.1001/archopht.122.4.477 15078664 10.1001/archopht.122.4.477

[CR11] Donda A, Mori L, Shamshiev A et al (2000) Locally inducible CD66a (CEACAM1) as an amplifier of the human intestinal T cell response. Eur J Immunol 30:2593–2603. 10.1002/1521-4141(200009)30:9<2593::AID-IMMU2593>3.0.CO;2-0 11009093 10.1002/1521-4141(200009)30:9<2593::AID-IMMU2593>3.0.CO;2-0

[CR12] Ergün S, Kilik N, Ziegeler G et al (2000) CEA-related cell adhesion molecule 1. Mol Cell 5:311–320. 10.1016/s1097-2765(00)80426-8 10882072 10.1016/s1097-2765(00)80426-8

[CR13] Ford KM, Saint-Geniez M, Walshe T et al (2011) Expression and role of VEGF in the adult retinal pigment epithelium. Invest Ophthalmol Vis Sci 52:9478–9487. 10.1167/iovs.11-8353 22058334 10.1167/iovs.11-8353 PMC3250352

[CR14] Götz L, Rueckschloss U, Ergün S, Kleefeldt F (2024) CEACAM1 in vascular homeostasis and inflammation. Eur J Clin Invest 54(Suppl 2):e14345. 10.1111/eci.14345 39674877 10.1111/eci.14345 PMC11646292

[CR15] Götz L, Rueckschloss U, Reimer A et al (2025) Vascular inflammaging: endothelial CEACAM1 expression is upregulated by TNF-α via independent activation of NF-κB and β-catenin signaling. Aging Cell 24:e14384. 10.1111/acel.14384 39434463 10.1111/acel.14384 PMC11822634

[CR16] Gray-Owen SD, Blumberg RS (2006) CEACAM1: contact-dependent control of immunity. Nat Rev Immunol 6:433–446. 10.1038/nri1864 16724098 10.1038/nri1864

[CR17] Guyer DR, Fine SL, Maguire MG et al (1986) Subfoveal choroidal neovascular membranes in age-related macular degeneration. Visual prognosis in eyes with relatively good initial visual acuity. Arch Ophthalmol 104:702–705. 10.1001/archopht.1986.01050170092029 2423062 10.1001/archopht.1986.01050170092029

[CR18] Hammarström S (1999) The carcinoembryonic antigen (CEA) family: structures, suggested functions and expression in normal and malignant tissues. Semin Cancer Biol 9:67–81. 10.1006/scbi.1998.0119 10202129 10.1006/scbi.1998.0119

[CR19] Ieda J, Yokoyama S, Tamura K et al (2011) Re-expression of CEACAM1 long cytoplasmic domain isoform is associated with invasion and migration of colorectal cancer. Int J Cancer 129:1351–1361. 10.1002/ijc.26072 21413011 10.1002/ijc.26072

[CR20] Kang W-Y, Chen W-T, Wu M-T, Chai C-Y (2007) The expression of CD66a and possible roles in colorectal adenoma and adenocarcinoma. Int J Colorectal Dis 22:869–874. 10.1007/s00384-006-0247-x 17143599 10.1007/s00384-006-0247-x

[CR21] Kilic N, Oliveira-Ferrer L, Wurmbach J-H et al (2005) Pro-angiogenic signaling by the endothelial presence of CEACAM1. J Biol Chem 280:2361–2369. 10.1074/jbc.M409407200 15536067 10.1074/jbc.M409407200

[CR22] Kim WM, Huang Y-H, Gandhi A, Blumberg RS (2019) CEACAM1 structure and function in immunity and its therapeutic implications. Semin Immunol 42:101296. 10.1016/j.smim.2019.101296 31604530 10.1016/j.smim.2019.101296 PMC6814268

[CR23] Kleefeldt F, Bömmel H, Broede B et al (2019) Aging-related carcinoembryonic antigen-related cell adhesion molecule 1 signaling promotes vascular dysfunction. Aging Cell 18:e13025. 10.1111/acel.13025 31389127 10.1111/acel.13025 PMC6826129

[CR24] Kurihara T, Westenskow PD, Bravo S et al (2012) Targeted deletion of Vegfa in adult mice induces vision loss. J Clin Invest 122:4213–4217. 10.1172/JCI65157 23093773 10.1172/JCI65157 PMC3484459

[CR25] Lange CAK, Luhmann UFO, Mowat FM et al (2012) Von Hippel-Lindau protein in the RPE is essential for normal ocular growth and vascular development. Development 139:2340–2350. 10.1242/dev.070813 22627278 10.1242/dev.070813 PMC3367444

[CR26] Lauke H (2004) Expression of carcinoembryonic antigen-related cell adhesion molecule-1 (CEACAM1) in normal human Sertoli cells and its up-regulation in impaired spermatogenesis. Mol Hum Reprod 10:247–252. 10.1093/molehr/gah020 14985475 10.1093/molehr/gah020

[CR27] Leung N, Turbide C, Olson M et al (2006) Deletion of the carcinoembryonic antigen-related cell adhesion molecule 1 (Ceacam1) gene contributes to colon tumor progression in a murine model of carcinogenesis. Oncogene 25:5527–5536. 10.1038/sj.onc.1209541 16619040 10.1038/sj.onc.1209541

[CR28] Ling Y, Wang J, Wang L et al (2015) Roles of CEACAM1 in cell communication and signaling of lung cancer and other diseases. Cancer Metastasis Rev 34:347–357. 10.1007/s10555-015-9569-x 26081722 10.1007/s10555-015-9569-x

[CR29] Love MI, Huber W, Anders S (2014) Moderated estimation of fold change and dispersion for RNA-seq data with DESeq2. Genome Biol 15:550. 10.1186/s13059-014-0550-8 25516281 10.1186/s13059-014-0550-8 PMC4302049

[CR30] Ludewig P, Sedlacik J, Gelderblom M et al (2013) Carcinoembryonic antigen-related cell adhesion molecule 1 Inhibits MMP-9–Mediated blood–brain–barrier breakdown in a mouse model for ischemic stroke. Circ Res 113:1013–1022. 10.1161/CIRCRESAHA.113.301207 23780386 10.1161/CIRCRESAHA.113.301207

[CR31] Ludewig P, Flachsbarth K, Wegscheid C et al (2014) CEACAM1 confers resistance toward oxygen-induced vessel damage in a mouse model of retinopathy of prematurity. Invest Ophthalmol Vis Sci 55:7950–7960. 10.1167/iovs.13-13403 25406283 10.1167/iovs.13-13403

[CR32] Marini F, Binder H (2019) PcaExplorer: an R/Bioconductor package for interacting with RNA-seq principal components. BMC Bioinformatics 20:331. 10.1186/s12859-019-2879-1 31195976 10.1186/s12859-019-2879-1 PMC6567655

[CR33] Moller MJ, Kammerer R, Grunert F, von Kleist S (1996) Biliary glycoprotein (BGP) expression on T cells and on a natural-killer-cell sub-population. Int J Cancer 65:740–745. 10.1002/(SICI)1097-0215(19960315)65:6<740::AID-IJC5>3.0.CO;2-Z 8631584 10.1002/(SICI)1097-0215(19960315)65:6<740::AID-IJC5>3.0.CO;2-Z

[CR34] Muturi HT, Ghadieh HE, Abdolahipour R et al (2023) Loss of CEACAM1 in endothelial cells causes hepatic fibrosis. Metabolism 144:155562. 10.1016/j.metabol.2023.155562 37088122 10.1016/j.metabol.2023.155562 PMC10330196

[CR35] Oldham MC, Langfelder P, Horvath S (2012) Network methods for describing sample relationships in genomic datasets: application to Huntington’s disease. BMC Syst Biol 6:63. 10.1186/1752-0509-6-63 22691535 10.1186/1752-0509-6-63 PMC3441531

[CR36] Patro R, Duggal G, Love MI et al (2017) Salmon provides fast and bias-aware quantification of transcript expression. Nat Methods 14:417–419. 10.1038/nmeth.4197 28263959 10.1038/nmeth.4197 PMC5600148

[CR37] Richardson KC, Jarett L, Finke EH (1960) Embedding in epoxy resins for ultrathin sectioning in electron microscopy. Stain Technol 35:313–323. 10.3109/10520296009114754 13741297 10.3109/10520296009114754

[CR38] Ritchie ME, Phipson B, Wu D et al (2015) limma powers differential expression analyses for RNA-sequencing and microarray studies. Nucleic Acids Res 43:e47. 10.1093/nar/gkv007 25605792 10.1093/nar/gkv007 PMC4402510

[CR39] Rovituso DM, Scheffler L, Wunsch M et al (2016) CEACAM1 mediates B cell aggregation in central nervous system autoimmunity. Sci Rep 6:29847. 10.1038/srep29847 27435215 10.1038/srep29847 PMC4951702

[CR40] Rueckschloss U, Kuerten S, Ergün S (2016) The role of CEA-related cell adhesion molecule-1 (CEACAM1) in vascular homeostasis. Histochem Cell Biol 146:657–671. 10.1007/s00418-016-1505-9 27695943 10.1007/s00418-016-1505-9

[CR41] Saint-Geniez M, Kurihara T, Sekiyama E et al (2009) An essential role for RPE-derived soluble VEGF in the maintenance of the choriocapillaris. Proc Natl Acad Sci U S A 106:18751–18756. 10.1073/pnas.0905010106 19841260 10.1073/pnas.0905010106 PMC2774033

[CR42] Sawa H, Kamada K, Sato H et al (1994) C-CAM expression in the developing rat central nervous system. Brain Res Dev Brain Res 78:35–43. 10.1016/0165-3806(94)90006-x 8004772 10.1016/0165-3806(94)90006-x

[CR43] Schlecht A, Leimbeck SV, Jägle H et al (2017) Deletion of endothelial transforming growth factor-β signaling leads to choroidal neovascularization. Am J Pathol 187:2570–2589. 10.1016/j.ajpath.2017.06.018 28823871 10.1016/j.ajpath.2017.06.018

[CR44] Schlecht A, Zhang P, Wolf J et al (2020) Secreted phosphoprotein 1 expression in retinal mononuclear phagocytes links murine to human choroidal neovascularization. Front Cell Dev Biol 8:618598. 10.3389/fcell.2020.618598 33585455 10.3389/fcell.2020.618598 PMC7876283

[CR45] Schlecht A, Thien A, Wolf J et al (2021) Immunosenescence in choroidal neovascularization (CNV)-transcriptional profiling of naïve and CNV-associated retinal myeloid cells during aging. Int J Mol Sci 22:13318. 10.3390/ijms222413318 34948115 10.3390/ijms222413318 PMC8707893

[CR46] Soneson C, Love MI, Robinson MD (2015) Differential analyses for RNA-seq: transcript-level estimates improve gene-level inferences. F1000Res 4:1521. 10.12688/f1000research.7563.2 26925227 10.12688/f1000research.7563.1 PMC4712774

[CR47] Stephens M (2017) False discovery rates: a new deal. Biostatistics 18:275–294. 10.1093/biostatistics/kxw041 27756721 10.1093/biostatistics/kxw041 PMC5379932

[CR48] Sulzbacher F, Pollreisz A, Kaider A et al (2017) Identification and clinical role of choroidal neovascularization characteristics based on optical coherence tomography angiography. Acta Ophthalmol 95:414–420. 10.1111/aos.13364 28133946 10.1111/aos.13364

[CR49] Taguchi F, Hirai-Yuki A (2012) Mouse hepatitis virus receptor as a determinant of the mouse susceptibility to MHV infection. Front Microbiol 3. 10.3389/fmicb.2012.00068 10.3389/fmicb.2012.00068 PMC3285771 22375141

[CR50] Thöm I, Schult-Kronefeld O, Burkholder I et al (2009) Expression of CEACAM-1 in pulmonary adenocarcinomas and their metastases. Anticancer Res 29:249–254 19331157

[CR51] Wang X, Abraham S, McKenzie JAG et al (2013) LRG1 promotes angiogenesis by modulating endothelial TGFß signalling. Nature 499. 10.1038/nature12345 10.1038/nature12345 PMC3836402 23868260

[CR52] Williams RK, Jiang GS, Holmes KV (1991) Receptor for mouse hepatitis virus is a member of the carcinoembryonic antigen family of glycoproteins. Proc Natl Acad Sci U S A 88:5533–5536. 10.1073/pnas.88.13.5533 1648219 10.1073/pnas.88.13.5533 PMC51911

[CR53] Wolf A, Herb M, Schramm M, Langmann T (2020) The TSPO-NOX1 axis controls phagocyte-triggered pathological angiogenesis in the eye. Nat Commun 11:2709. 10.1038/s41467-020-16400-8 32483169 10.1038/s41467-020-16400-8 PMC7264151

[CR54] Wong TY, Wong T, Chakravarthy U et al (2008) The natural history and prognosis of neovascular age-related macular degeneration. Ophthalmology 115(1):116. 10.1016/j.ophtha.2007.03.008 17675159 10.1016/j.ophtha.2007.03.008

[CR55] Yang C, He P, Liu Y et al (2015) Down-regulation of CEACAM1 in breast cancer. Acta Biochim Biophys Sin (Shanghai) 47:788–794. 10.1093/abbs/gmv075 26341981 10.1093/abbs/gmv075

[CR56] Zhang P, Schlecht A, Wolf J et al (2021) The role of interferon regulatory factor 8 for retinal tissue homeostasis and development of choroidal neovascularisation. J Neuroinflammation 18:215. 10.1186/s12974-021-02230-y 34544421 10.1186/s12974-021-02230-y PMC8454118

