# Supplementary figures and images for: Deletion of CEACAM1 does not affect retinal and choroidal morphology or transcriptome
Source: Cell Tissue Res. 2026 Jun 26;405(1):4. doi: 10.1007/s00441-026-04087-0 (PMC13309465; doi:10.1007/s00441-026-04087-0)

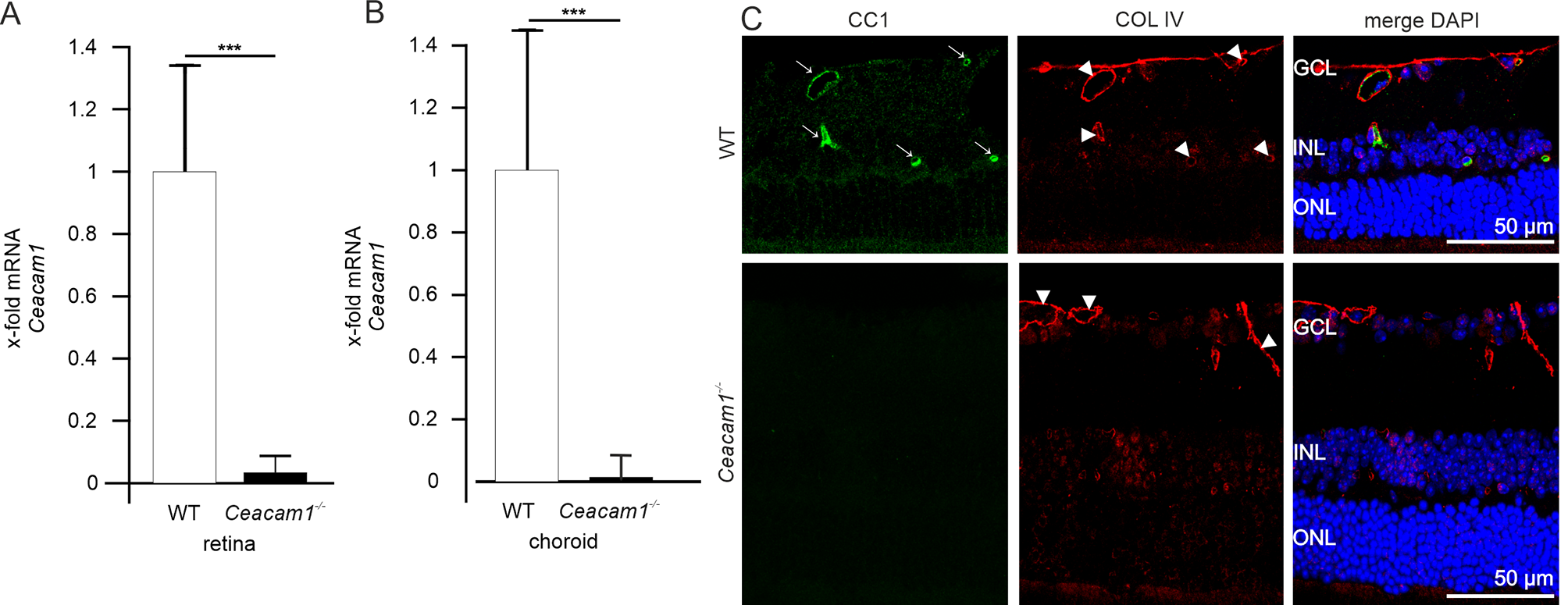

Supplement: Supplementary file 1 — Supplementary Figure 1: Deletion of CC1 in retina and choroid. A, B: Retinal (A) and choroidal (B) mRNA expression of Ceacam1 in 2-4 month-old wild type and Ceacam1-/- mice. Data are means ± SD. (retina: wild type n = 3 and Ceacam1-/- n = 5; choroid: wild type n = 6 and Ceacam1-/- n = 6), *** p ≤ 0.001. C : CC1 (green, arrows) and COL IV (red, arrowheads) double labelling of 3 months old wild type and Ceacam1-/- eyes. Nuclei are DAPI-stained (blue). GCL = ganglion cell layer, INL = inner nuclear layer, ONL = outer nuclear layer. (PNG 689 KB) [file 441_2026_4087_Fig5_ESM.png]

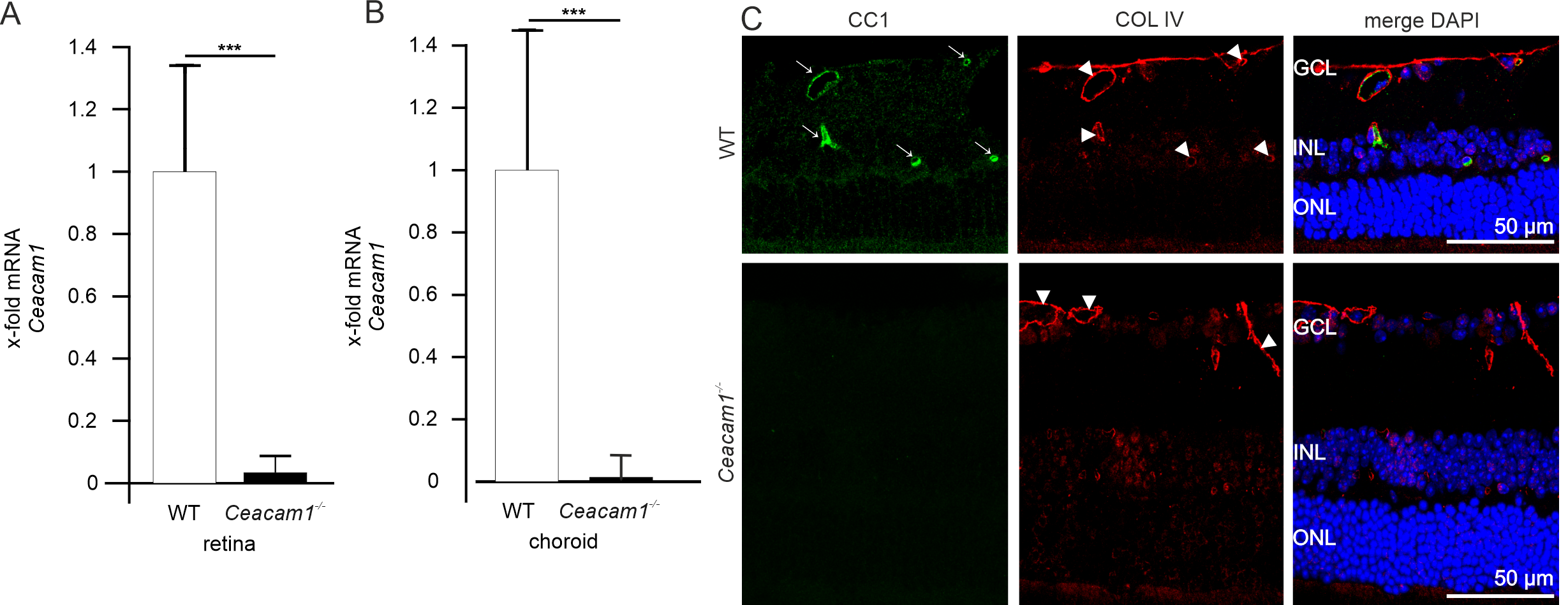

Supplement: Supplementary file 2 — High Resolution Image (TIF 6.68 MB) [file 441_2026_4087_MOESM1_ESM.tif]

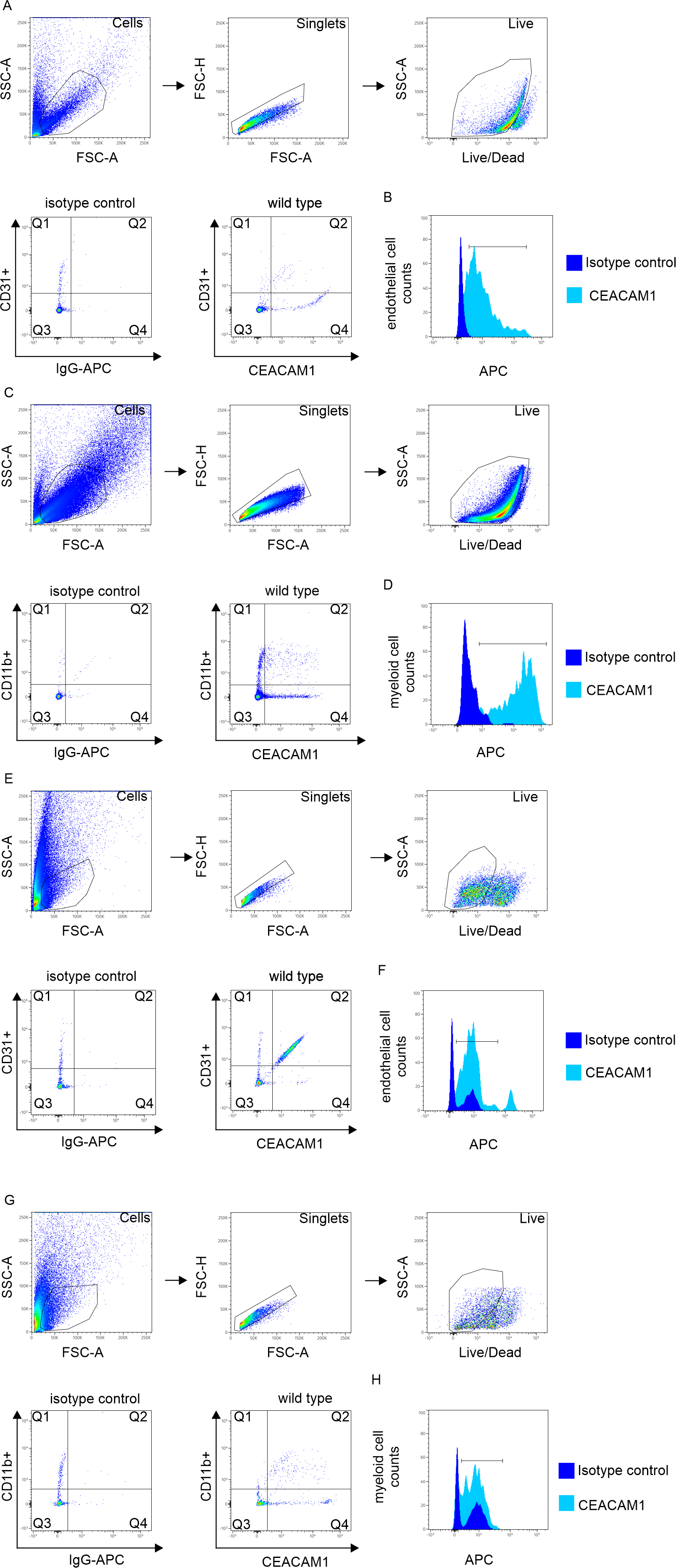

Supplement: Supplementary file 3 — Supplementary Figure 2: Representative FACS plots for gating strategy and isotype controls. A representative flow cytometry gating strategy for analyzing CEACAM1 expression in myeloid and endothelial cells of the retina (A-D) and choroid (E-H). First, cells were identified based on FSC-A/SSC-A, then singlets were selected using FSC-H/FSC-A, and live cells were gated using live/dead cell staining. Next, endothelial cells (CD31+) (A, E) or myeloid cells (CD11b+) (C, G) were identified, and CEACAM1 expression was analyzed in comparison to the respective isotype control. The histograms show the distribution of CEACAM1-positive endothelial (B, F) or myeloid cells (D, H). (PNG 674 KB) [file 441_2026_4087_Fig6_ESM.png]

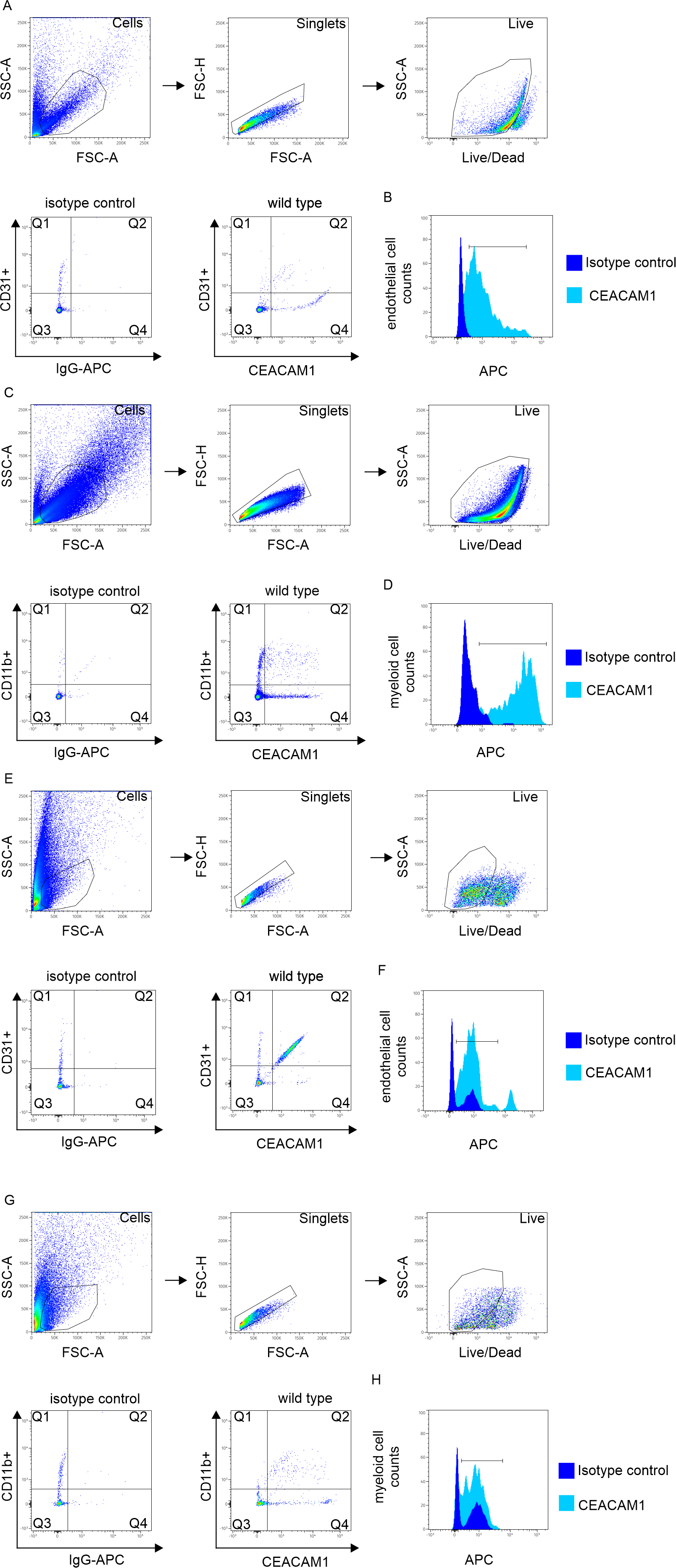

Supplement: Supplementary file 4 — High Resolution Image (TIF 17.1 MB) [file 441_2026_4087_MOESM2_ESM.tif]
